# A tribute to Dr Naftali Tuyoleni Hamata

**DOI:** 10.4102/phcfm.v15i1.4120

**Published:** 2023-05-31

**Authors:** Daniel O Ashipala, Lukas K. Katenda

**Affiliations:** 1 Department of General Nursing Sciences, Faculty of Health Sciences and Veterinary Medicine, University of Namibia, Rundu, Namibia; 2 Reformed Evangelical Anglican of Namibia, Bethlehem Church, Anglican Reach-Namibia, Windhoek, Namibia

The first day of March 2023 will be remembered not only by the family of Dr Naftali Tuyoleni Hamata but also by the Namibian health fraternity and the many people he touched with his kind heart and hand. On that day, Dr Hamata passed into the eternal realm at the age of 78 years.

Dr Hamata was fortunate to be born to his admirable parents, Henock Hamata and Saara Nhinda-Hamata, who ensured that their son could attend the Lutheran mission school in Engela for the entirety of his school career, despite not being fashionable at the time.^1^ The time Dr Hamata spent at school yielded great dividends, including his acceptance to university to study medicine. Upon successfully matriculating from high school, Dr Hamata was accepted into the University of Natal (now know as the University of KwaZulu-Natal), South Africa, from which he graduated with a Bachelor of Medicine and a Bachelor of Surgery (MBChB) in 1976. Credit must also be given to the Evangelical Lutheran Church in Namibia (ELCIN), which under the leadership of Bishop Leonard Auala noticed the young Hamata’s skills and attributes and arranged a scholarship for his education.^1,2,3^

In 1984, Dr Hamata was accorded another opportunity to study further and specialise in the area of gynaecology and obstetrics. Upon his return from university, he worked at Onandjokwe Lutheran Hospital before moving to Oshakati State Hospital, where he took on the role of medical superintendent in 1990. He was later promoted to Director of Health for the north-western regions in 1997.^2,4^

Throughout his illustrious career, Dr Hamata always made himself available to young student nurses, whether in his consulting room or in the operating room, often teaching them as a procedure was underway. Dr Hamata fondly came to be known as ‘Tatekulu’ during his time at Oshakati State Hospital.

In addition to being a practicing gynaecologist, philanthropist and freedom fighter, Dr Hamata was appointed as a Special Advisor to the Minister of Health. He also played a domestic role as a beloved husband and father.^2,4,5^ Dr Hamata served with such honour that the Head of State, Dr Hage G. Geingob, conferred on him a state funeral and military parade, which took place on 18 March 2023.^3^
